# Selection of a stealthy and harmful attack function in discrete event systems

**DOI:** 10.1038/s41598-022-19737-w

**Published:** 2022-09-29

**Authors:** Qi Zhang, Carla Seatzu, Zhiwu Li, Alessandro Giua

**Affiliations:** 1 School of Electro-Mechanical Engineering, Xidian University, Xi’an, 710071 China; 2 Department of Electrical and Electronic Engineering, University of Cagliari, 09123 Cagliari, Italy; 3 Institute of Systems Engineering, Macau University of Science and Technology, Macau, 999078 China

**Keywords:** Computer science, Computational science

## Abstract

In this paper we consider the problem of joint state estimation under attack in partially-observed discrete event systems. An operator observes the evolution of the plant to evaluate its current states. The attacker may tamper with the sensor readings received by the operator inserting dummy events or erasing real events that have occurred in the plant with the goal of preventing the operator from computing the correct state estimation. An attack function is said to be harmful if the state estimation consistent with the correct observation and the state estimation consistent with the corrupted observation satisfy a given misleading relation. On the basis of an automaton called joint estimator, we show how to compute a supremal stealthy joint subestimator that allows the attacker to remain stealthy, no matter what the future evolution of the plant is. Finally, we show how to select a stealthy and harmful attack function based on such a subestimator.

## Introduction

The problem of cyber attacks in discrete event systems (DESs) has been addressed by several authors^1–3^. An attacker may insert (erase) sensor readings received by the supervisor (sensor attacks)^4,5^, and may corrupt the control actions of the supervisor (actuator attacks)^6–8^. In such a case, the specification imposed by the supervisor may be violated. Typical examples on cyber attacks of discrete event systems are as follows.

Carvalho et al.^9^ use a diagnoser-based approach to detect the attacker. Once it is detected, the supervisor will disable all the controllable events to keep the system safe. Lin and Su^10^ investigate the problem of synthesizing the covert attackers considering sensor replacement attacks, actuator enablement attacks, and actuator disablement attacks. They transform such a problem into the Ramadge-Wonham supervisor synthesis problem so that existing techniques can be used.

Meira-Góes et al.^11^ study the problem of stealthy sensor attacks at the supervisory control layer. They define a structure called *insertion-deletion attack* structure that characterizes the interaction between the supervisor and the environment (includes the plant and the attacker). Such a structure can be used to synthesize three different kinds of attack policies: *unbounded deterministic* attack, *bounded deterministic* attack, and *interruptible* attack. Meira-Góes et al.^12^ develop a structure called *solution-arena* ${\mathscr {A}}^{sup}$ that contains all the *robust* supervisors against sensor deception attacks. Lima et al.^13^ introduce the notion of network attack security. If the plant is network attack secure, then they present an algorithm to compute a security supervisor that can prevent the plant from reaching the unsafe state.

Alves et al.^14^ consider the problem of sensor attacks in DESs, where an attacker can corrupt the supervisor observation by inserting or erasing symbols from a specific set, but we do not know which symbol will be tampered during an attack. They present a new method to verify if a given language is *P-observable* in terms of a new observability condition. The problem of stealthy attacker synthesis has been addressed by Lin et al.^15^, where the considered attacks include the sensor replacement attack and the actuator disablement attack. The authors assume that the attacker does not know the model of the supervisor, but can record a finite observation of the closed-loop system. Meira-Góes and Lafortune^16^ introduce a new defense strategy against sensor attacks called moving target defense paradigm, i.e., a plant is controlled by a set of supervisors, and only one supervisor is active at a certain time. They provide a necessary and sufficient condition for the existence of supervisors and a switching mechanism between them such that the specifications of safety, liveness, and maximal permissiveness can be enforced.

There are also many other works that consider the problem of cyber attacks in different settings. Li et al.^17^ propose the problem of actuator enablement attacks in networked control systems, where control delays of the supervisor may occur. The problems of optimal multi-objective attack policies and mitigation of attacks are discussed in the context of probabilistic discrete event systems^18,19^. The problem of sensor attacks for distributed control systems has been investigated^20,21^. The issue of attack-resilient supervisory control is studied by Wang et al.^22,23^, where the attacker is modeled as finite state transducers. You et al.^24,25^ consider the problem of supervisory control under sensor attacks using Petri nets. Wang et al.^26^ consider the issue of supervisory control under attack including sensor attacks and actuator attacks, where the adopted model is labeled Petri nets. Finally, the problem of performance safety enforcement is proposed in the context of timed Petri nets and stochastic Petri nets, respectively^27,28^.

In this paper we study the problem of *joint state estimation under attack* in partially-observed discrete event systems. Assume that the plant and the operator are connected via a network that transmits the sensor readings of the events that have occurred in the plant. The operator observes the evolution of the plant with the objective of identifying its current state. An attacker, which has a full knowledge of the plant, may corrupt the operator’s observation by inserting fake events and erasing real events that have occurred in the plant so that the correct state estimation of the operator is compromised. In addition, we assume that the attacker needs to be stealthy, i.e., the operator should not be able to detect that the plant is under attack.

Given a plant $G=(X, E, \delta , x_0)$, assume that a word $\sigma \in E^*$ is generated. If there is no attack, the operator observes the word $P(\sigma )=s \in E_o^*$, where *P* is the *natural projection* on the set of observable events $E_o$, and computes the state estimate ${\mathscr {C}}(s)\subseteq X$, namely the *set of states consistent with the observation* *s*. Nevertheless, if an attacker gets involved changing the word *s* into a corrupted observation $s' \in E_o^*$, then the operator will construct a wrong state estimate ${\mathscr {C}}(s') \subseteq X$. To formalize such a problem, we introduce the notion of *misleading relation* ${\mathscr {R}} \subseteq 2^X \times 2^X$, and an attack is said to be *harmful* if $({\mathscr {C}}(s),{\mathscr {C}}(s')) \in {\mathscr {R}}$.

The motivation of introducing the misleading relation ${\mathscr {R}}$ can be explained as follows. Assume that a plant $G=(X, E, \delta , x_0)$ contains a set of *critical states* $X_{cr} \subseteq X$. An operator observes the plant evolution with the goal of establishing if a critical state $x \in X_{cr}$ is reached so that it should activate protective actions that are essential for the safety of the plant. However, when a critical state is reached, if the attacker corrupts the operator’s observation making it believe that a non-critical state is reached, then no protective action is activated, and damages are caused to the system.

In Zhang et al.^29^, an automaton called *joint estimator*, which allows one to establish for each attack word, the joint state estimation of the attacker and of the operator, is constructed. This paper extends the work of Zhang et al.^29^. First, we show how to trim the joint estimator obtaining the *supremal stealthy joint subestimator* ${\widehat{A}}$ that contains all the stealthy attacks. Then, we give a procedure to compute a stealthy attack function $f^s$ from ${\widehat{A}}$. Finally, we discuss the existence of a stealthy and harmful attack function w.r.t. a misleading relation ${\mathscr {R}}$.

We point out that, in the framework of discrete event systems, most of the existing works on cyber attacks consider such a problem at the supervisory control layer. However, in this paper the problem of joint state estimation under attack is proposed at the observation layer. Among the previous mentioned literature, Meira-Góes et al.^11^ inspired us most. However, there are three fundamental differences between the two works.

1. In terms of problem setting, we do not address a problem of supervisory control under attack, as in Meira-Góes et al.^11^, but a problem of state estimation under attack. In the framework of supervisory control, one assumes that a supervisor has been designed to enforce a given specification. When the plant is under attack, the goal is that of understanding if the action of the attacker may mislead the supervisor so that the controlled system violates the specification and a supervisor which is robust under attack is eventually constructed. In the framework of state estimation, one assumes that an observer has been designed to allow an operator to reconstruct the state of a monitored plant from its observed outputs. When the plant is under attack, the goal is that of understanding how the action of the attacker may mislead the operator to make a wrong estimation.
2. In terms of the structures that provide the solution, in Meira-Góes et al.^11^ a bipartite structure called *insertion-deletion attack structure* is used to solve the problem of supervisory control under attack. In our approach, an automaton, called *joint estimator* is adopted to solve the problem of state estimation under attack. The two structures are rather different.
3. In terms of the synthesis of attack functions, in Meira-Góes et al.^11^ an attack function that has the shortest path to reach an unsafe state is synthesized. While in this work, a harmful attack function w.r.t. a misleading relation ${\mathscr {R}}$ that does not stop at a *preempting state* to remain stealthy is selected.

The remainder of the paper is organized as follows. “Preliminaries” section presents the basic concepts of discrete event systems (e.g. *automata* and *observer*). “Attack model” section introduces the definition of an attack function, and the attack model. “Attacker observer, operator observer, and joint estimator” section recalls the definitions of *attacker observer*, *operator observer*, and *joint estimator* from Zhang et al.^29^. “Problem statement” section provides the problem statement in terms of the harmfulness and stealthiness of an attack function. “Supremal stealthy joint subestimator” section details how to extract the supremal stealthy joint subestimator from a joint estimator. “Computing stealthy and harmful attack function” section discusses how to select a stealthy and harmful attack function on the basis of the supremal stealthy joint subestimator. “Conclusions and future work” section reports the conclusions and our possible future works in this topic.

## Preliminaries

An alphabet *E* is a finite and non empty set of symbols and a word defined over *E* is a string composed by its symbols. Given an alphabet *E*, we denote as $E^*$ the set of all finite words defined over *E*, including the empty word $\varepsilon$ that contains no symbol. As an example, let $E=\{a,b,c,d\}$. Then

$$\begin{aligned} E^*=\{\varepsilon ,a,b,c,d,aa,ab,ac,ad,ba,bb,bc,bd,ca,cb,cc,cd,da,db,dc,dd,aaa,...\}. \end{aligned}$$

Note that the set $E^*$ is countably infinite.

Let *D* be a set. The *power set* of *D*, denoted as $2^D$, is the set of all the subsets of *D*, i.e., $2^D=\{d \ | \ d \subseteq D\}$.

A *deterministic finite-state automaton* (DFA) is a four-tuple, denoted by $G=(X, E, \delta , x_0)$, where *X* is the set of *states*, *E* is an alphabet, $\delta : X \times E \rightarrow X$ is the *transition function*, i.e., $\delta (x,e)=x'$ means that at state *x*, there exists a transition labeled *e* yielding state $x'$, and $x_0$ is the *initial state*. The transition function can be extended to the domain $X \times E^*$, denoted by $\delta ^*: X \times E^* \rightarrow X$ such that $\delta ^*(x,\sigma e)=\delta (\delta ^*(x,\sigma ),e)$, where $\sigma \in E^*$. The *language generated* by *G* is defined by $L(G)=\{\sigma \in E^* \ | \ \delta ^*(x_0,\sigma )\text { is defined}\}$.

When automata are used to describe discrete event systems, the alphabet *E* represents the set of events that the system can generate. A word represents a particular evolution of the system and the generated language of the automaton represents the behavior of the system, i.e., the set of all its evolutions. The set of *active events* at state *x* is denoted as $\Gamma (x)=\{e \in E \ | \ \delta (x,e) \text { is defined}\}$. Given two *words* $\sigma _1, \sigma _2 \in L(G)$, we denote their *concatenation* $\sigma _1 \sigma _2$.

Due to the absence of sensors to record the occurrence of some events, not all the events are observable. As a result, the event set *E* is partitioned into two disjoint subsets: the set of *observable events* $E_o$ and the set of *unobservable events* $E_{uo}$.

Given two alphabets *E* and $E'$ such that $E' \subseteq E$. The *natural projection*^30^ on $E'$, $P_{E'}: E^* \rightarrow (E')^*$ is defined as follows:

1 $$\begin{aligned} P_{E'}(\varepsilon ):=\varepsilon \ , \ P_{E'}(\sigma e) := \left\{ \begin{array}{lcl} {P_{E'}(\sigma ) e} \ &{}\text {if}&{} \ e \in E', \\ {P_{E'}(\sigma )} \ &{}\text {if}&{} \ e \in E \setminus E'. \end{array} \right. \end{aligned}$$

In plain words, given a word $\sigma \in E^*$, the natural projection $P_{E'}$ removes from $\sigma$ events that do not belong to $E'$.

Given two DFA $G_1=(X_1, E_1, \delta _1, x_{01})$, $G_2=(X_2, E_2, \delta _2, x_{02})$, and set $E=E_1 \cup E_2$. The *concurrent composition* of $L(G_1)$ and $L(G_2)$ is defined by $L(G_1) \parallel L(G_2)=\{\sigma \in E^* \ | \ P_{E_1}(\sigma ) \in L(G_1), P_{E_2}(\sigma ) \in L(G_2)\}$.

For the sake of simplicity, we use $P: E^* \rightarrow E_o^*$ to denote the natural projection from $E^*$ to $E_o^*$. Correspondingly, the *inverse projection* $P^{-1}: E_o^* \rightarrow 2^{E^*}$ is defined by $P^{-1}(s)=\{\sigma \in E^*:P(\sigma )=s\}$. In words, the inverse projection $P^{-1}(s)$ returns the set of all words $\sigma \in E^*$ that produce the observation *s*.

Consider a partially-observed DFA $G=(X, E, \delta , x_0)$, and let $s \in P[L(G)]$ be an observation. In general there may exist more than one generated word $\sigma \in L(G)$ that produces observation *s*. The *set of words consistent with observation* *s* is defined as:

2 $$\begin{aligned} {\mathscr {S}}(s) = P^{-1}(s) \cap L(G) = \{ \sigma \in L(G) \mid P(\sigma ) = s \}, \end{aligned}$$

and denotes the set of all words $\sigma \in L(G)$ that produce observation *s*.

The *set of states consistent with observation* *s* is defined as:

3 $$\begin{aligned} {\mathscr {C}}(s) = \{ x \in X \mid (\exists \sigma \in {\mathscr {S}}(s)) \ \delta ^*(x_0,\sigma ) = x \}, \end{aligned}$$

and denotes the set of all states where the DFA may be when observation *s* is produced.

We also call ${\mathscr {C}}(s) \subseteq X$ the *state estimate* that corresponds to the observation $s \in E_o^*$. One may use the notion of *observer*^31^ to solve the problem of state estimation for a partially-observed DFA. In order to construct the observer, the definition of *unobservable reach* is first introduced.

The *unobservable reach* *UR*(*x*) of state $x \in X$ is defined by $UR(x) =\{x' \ | \ \exists \sigma \in E_{uo}^*, \ \delta ^*(x,\sigma )=x'\}$. In words, *UR*(*x*) is the set of states $x' \in X$ reached from state *x* executing an unobservable word $\sigma$. Such a definition is extended to a set of states $B \subseteq X$ as:

4 $$\begin{aligned} UR(B)=\bigcup \limits _{x\in B}UR(x). \end{aligned}$$

Let $G=(X, E, \delta , x_0)$ be a partially-observed DFA with $E_o \subseteq E$, its *observer* *Obs*(*G*) is still a DFA:

5 $$\begin{aligned} Obs(G) = (B,E_o,{\delta _{obs}},b_0), \end{aligned}$$

where $B\subseteq 2^X$ is the set of states, $E_o$ is the set of observable events, $\delta _{obs}: B\times E_o \rightarrow B$ is the transition function defined by:

6 $$\begin{aligned} \delta _{obs}(b,e_o):=\bigcup \limits _{x\in b}UR(\{ x' \mid \delta (x,e_o)=x'\}), \end{aligned}$$

and $b_0:=UR(x_0)$ is the initial state.

Let *G* be a partially-observed DFA with the observer $Obs(G)=(B,E_o,{\delta _{obs}},b_0)$. Given an observation $s \in P[L(G)]$, it holds that the set of states consistent with the observation *s* is ${\mathscr {C}}(s)=\delta _{obs}^*(b_0,s)$.

## Attack model

In this paper we adopt the attack model in Zhang et al.^29^, namely we assume that the attacker can insert dummy events and erase real events to interfere in the operator observation. To make the paper self-contained, in the following we recall some key definitions from Zhang et al.^29^.

**Figure 1:**
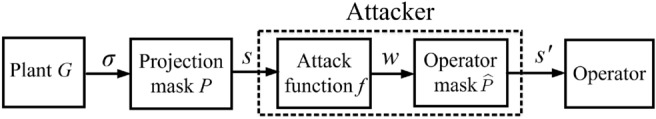
A plant *G* under attack.

As shown in Fig. 1, we assume that $\sigma \in E^*$ is a word generated by the plant *G* producing the *observed word* $s=P(\sigma )$. The attacker may tamper with such an observation inserting fake signals or erasing real signals. Therefore, the operator receives the *corrupted observation* $s' \in E_o$, and computes its state estimation according to it (neglect the internal structure of an attacker within the dotted lines for the moment). The goal of the attacker is to mislead the operator to construct the wrong state estimation.

Note that, in this paper, we assume that the attacker has a full knowledge of the plant, namely the attacker knows the model of the plant, and observes the plant with the same projection mask used by the operator, i.e., if the operator can (cannot) observe a certain event of the plant, then the attacker can (cannot) observe it. The above assumption is common to many works^2,9,11,13^ on cyber attacks in the framework of DES. We will consider the case in which the attacker only has a partial knowledge of the plant model in future work.

We assume that the plant contains a set of *compromised events* $E_{com} \subseteq E_o$, i.e., the set of events that could be inserted or erased by the attacker. Note that such a definition was first proposed by Meira-Góes et al.^11^, however, it has been slightly generalized in Zhang et al.^29^ partitioning $E_{com}$ into two subsets: the set of events that can be inserted, denoted as $E_{ins}$; the set of events that can be erased, denoted as $E_{era}$. Namely $E_{com}=E_{ins} \cup E_{era}$. We point out that, $E_{ins}$ and $E_{era}$ are not necessarily disjoint.

In order to distinguish the original events of the plant from those produced by the action of the attacker, we introduce two new alphabets $E_+$ and $E_-$. We denote as $E_+=\{e_+ \mid e \in E_{ins}\}$ the set of *inserted events*^11^, and $E_-=\{e_- \mid e \in E_{era}\}$ the set of *erased events*^11^. The occurrence of an event $e_+ \in E_+$ means that *e* does not occur but the attacker inserts a fake signal *e* to the operator observation. The occurrence of an event $e_- \in E_-$ implies that *e* has occurred in the plant but the attacker erases it. Finally, the *attack alphabet* is defined by $E_a=E_o \cup E_+ \cup E_-$. Note that by definition sets $E_o$, $E_+$, and $E_-$ are disjoint.

### Definition 1

Let *G* be a plant with set of compromised events $E_{com}=E_{ins} \cup E_{era}$, and $E_a=E_o \cup E_+ \cup E_-$ be the attack alphabet. An attacker can be defined as an attack function $f: P[L(G)] \rightarrow E_a^*$:

1. $$f(\varepsilon )\in E_+^*$$,
2. $$\forall se \in P(L(G))$$ such that $$s \in E_o^*$$: $$\left\{ \begin{array}{ll} f(se) \in f(s)\{e_-,e\}E_+^* &{}\text {if} \quad e \in E_{era}, \\ f(se) \in f(s) \{ e \} E_+^* &{}\text {if} \quad e \in E_o \setminus E_{era}. \end{array} \right.$$

$$\square$$

In plain words, condition (1) indicates that the attacker may insert any word in $E_+^*$ when no observable event is produced by the plant. Condition (2) means that if an event $e \in E_{era}$ happens, the attacker can either erase it or not, and then insert any word in $E_+^*$. Furthermore, the attacker may also insert any word in $E_+^*$ after the occurrence of an event in $E_o \setminus E_{era}$, which cannot be erased.

Due to the presence of an attack function, the augmented language of the plant is called *attack language*, defined as $L(f,G)=f(P[L(G)])$. We use *w* to denote a word in *L*(*f*, *G*), and call it *attack word*.

In order to characterize how the operator treats events in the attack alphabet $E_a$, we define the *operator mask* ${\widehat{P}}:E_a^* \rightarrow E_o^*$ as follows:

7 $$\begin{aligned} {\widehat{P}}(\varepsilon )=\varepsilon , \ {\widehat{P}}(w e')=\left\{ \begin{array}{lcl} {{\widehat{P}}(w)e} &{}\text {if}&{} e'=e \in E_o \ \vee e'=e_+ \in E_+, \\ {{\widehat{P}}(w)} &{}\text {if}&{} e'=e_- \in E_-. \end{array} \right. \end{aligned}$$

Given an attack word $w \in E_a^*$, the operator mask reduces *w* into a word in $E_o^*$. In fact, the operator cannot distinguish event $e \in E_o$ from the corresponding event $e_+$, and it cannot observe events in $E_-$.

The internal structure of an attacker is depicted in Fig. 1 within the dotted lines. First, the attack function changes an observed word $s \in E_o^*$ into an attack word $w \in E_a^*$. Then, the operator mask reduces *w* to a corrupted observation $s' \in E_o^*$.

## Attacker observer, operator observer, and joint estimator

In this section, we review the notions of attacker observer, operator observer, and joint estimator, which were first proposed by Zhang et al.^29^.

### Attacker observer

Given a plant $G=(X, E, \delta , x_0)$, the attacker observer is constructed based on the observer of the plant $Obs(G)=(B,{E_o},{\delta _{obs}},b_0)$. The attacker observer generates all the attack words on the attack alphabet $E_a$ resulting from the attack function, and provides the state estimation of the attacker according to the attack words. One can use Algorithm 1 in Zhang et al.^29^ to compute the attacker observer.

#### Definition 2

Consider a plant $G=(X, E, \delta , x_0)$ with the observer $Obs(G) = (B,E_o,{\delta _{obs}},b_0)$. Let $E_{ins} \subseteq E_o$ be the set of events that can be inserted by the attacker, $E_{era} \subseteq E_o$ be the set of events that can be erased by the attacker, and $E_a=E_o \cup E_+ \cup E_-$ be the attack alphabet. The attacker observer is defined as $Obs_{att}(G) = (B,E_a,\delta _{att},b_0)$, where the transition function $\delta _{att}$ is defined as follows:

8 $$\begin{aligned} \left\{ \begin{array}{lcl} \forall b \in B, \ \forall e \in E_o, \ \delta _{att}(b,e)=\delta _{obs}(b,e),\\ \forall b \in B, \ \forall e \in E_{era}, \ \delta _{att}(b,e_-)=\delta _{att}(b,e), \\ \forall b \in B, \ \forall e \in E_{ins}, \ \delta _{att}(b,e_+)=b. \end{array} \right. \end{aligned}$$

$$\square$$

According to Definition 2, first, for all the states $b \in B$, and for all the observable events $e \in E_o$, we impose $\delta _{att}(b,e)=\delta _{obs}(b,e)$, i.e., the transition function of the attacker observer is initialized at the transition function of the observer of the plant. Then, for each observable event $e \in E_{era}$, namely for each event whose observation can be erased, we add the transition $\delta _{att}(b,e_-)=\delta _{att}(b,e)$. In fact, the attacker knows that $e_- \in E_-$ is an erased event that has occurred in the plant, and thus the attacker updates its state in the same way it does when event $e \in E_{era}$ occurs. Finally, for all the events $e \in E_{ins}$, we add self-loops labeled $e_+$ at all the states of $Obs_{att}(G)$. In fact, the attacker knows that an event in $E_+$ is a dummy event inserted by itself, thus the attacker does not update its state when $e_+$ occurs.

In the following, given a plant *G*, we denote as ${\mathscr {F}}$ the *set of attack functions*, and define $L({\mathscr {F}},G)=\bigcup \limits _{f \in {\mathscr {F}}}f(P[L(G)])$ the *union of all the attack languages*, where *P* is the natural projection. The following proposition sketches the language and the transition function of an attacker observer.

#### Proposition 3

^29^ *Consider a plant* *G* *with its observer* $Obs(G) = (B,{E_o},{\delta _{obs}},b_0)$. *Let* ${\mathscr {F}}$ *be the set of attack functions, and* $Obs_{att}(G) = (B,E_a,\delta _{att},b_0)$ *be the attacker observer constructed using Algorithm 1 in Zhang et al.*^29^. *The following statements hold*:

- $$L[Obs_{att}(G)]=L({{\mathscr {F}}},G)$$;
- $$\forall s \in P[L(G)]$$, $$\forall f \in {{\mathscr {F}}}$$ *with* $$w=f(s) \in E_a^*$$: $$\delta _{att}^*(b_0,w)=\delta _{obs}^*(b_0,s).$$ $$\Box$$

The formal proof of this proposition can be found in Zhang et al.^29^ (Proposition 1 therein). The language of an attacker observer $L[Obs_{att}(G)]$ is equal to the union of all the attack languages $L({{\mathscr {F}}},G)$. According to the construction of the attacker observer, $L[Obs_{att}(G)]$ contains all the words that correspond to the original behavior of the plant *G*, the insertion of events in $E_+$, and the erasure of events in $E_-$.

From the initial state $b_0$, the state reached in $Obs_{att}(G)$ by executing $w=f(s)$ is equal to the state reached in *Obs*(*G*) by executing *s*. This follows from the fact that events in $E_+$ are self-loops in $Obs_{att}(G)$, and $Obs_{att}(G)$ updates its states the same way in case of event $e \in E_{era}$ and the corresponding event $e_-$.

### Operator observer

Given a plant $G=(X, E, \delta , x_0)$, the operator observer is built based on the observer of the plant $Obs(G)=(B,{E_o},{\delta _{obs}},b_0)$. The operator observer characterizes the state estimation of an operator according to the attack words $w \in E_a^*$. Such a structure generates two sets of words. The first set contains all the words that keep the attacker stealthy, and the second set includes all the words that reveal the presence of an attacker. In the operator observer, the second set of words lead to a fake state, denoted as $b_\emptyset$. When such a state is reached, the operator realizes that the plant is under attack. The operator observer can be computed using Algorithm 2 in Zhang et al.^29^.

#### Definition 4

Consider a plant $G=(X, E, \delta , x_0)$ with observer $Obs(G) = (B,E_o,{\delta _{obs}},b_0)$. Let $E_{ins} \subseteq E_o$ be the set of events that can be inserted by the attacker, $E_{era} \subseteq E_o$ be the set of events that can be erased by the attacker, and $E_a=E_o \cup E_+ \cup E_-$ be the attack alphabet. The operator observer is defined by $Obs_{opr}(G) = (B_{opr},E_a,\delta _{opr},b_0)$, where $B_{opr}=B \cup b_\emptyset$, and the transition function $\delta _{opr}$ is defined as:

9 $$\begin{aligned} \left\{ \begin{array}{lcl} \forall b \in B, \ \forall e \in E_o, \ \delta _{opr}(b,e)=\delta _{obs}(b,e),\\ \forall b \in B, \ \forall e \in E_{ins}, \ \delta _{opr}(b,e_+)=\delta _{opr}(b,e), \\ \forall b \in B, \ \forall e \in E_{era}, \ \delta _{opr}(b,e_-)=b, \\ \forall b \in B, \ \forall e \in E_a, \text { if } \delta _{opr}(b,e) \text { is not defined, then } \delta _{opr}(b,e)=b_\emptyset . \end{array} \right. \end{aligned}$$

$$\square$$

In accordance with Definition 4, first, for all the states $b \in B$, and for all the observable events $e \in E_o$, we impose $\delta _{opr}(b,e)=\delta _{obs}(b,e)$, i.e., the transition function of $Obs_{opr}(G)$ is initialized at the transition function of *Obs*(*G*). Then, for each observable event $e \in E_{ins}$, namely for each event whose observation can be inserted, we add the transition $\delta _{opr}(b,e_+)=\delta _{opr}(b,e)$. This occurs because the operator cannot distinguish an event in $E_{ins}$ from the corresponding event in $E_+$. Furthermore, for all the events $e \in E_{era}$, we add self-loops labeled $e_-$ at all the states of $Obs_{opr}(G)$. This happens because the operator cannot observe events in $E_-$. Finally, for all the states $b \in B$ and for all the events $e \in E_a$, if $\delta _{opr}(b,e)$ is not defined, then we impose $\delta _{opr}(b,e)=b_\emptyset$. In this way, for all the states $b \in B$ and for all the events $e \in E_a$, $\delta _{opr}(b,e)$ is defined. On the contrary, for state $b_\emptyset$, no transition function is defined.

In the following, given a plant *G*, we use $W_s=\{w \in E_a^* \ | \ {\widehat{P}}(w) \in P(L(G))\}$ to denote the *set of stealthy words*, and $W_e=\{we \in E_a^* \ | \ w \in W_s, e \in E_a, w e \not \in W_s\}$ to denote the *set of exposing words*, where *P* is the natural projection, and ${\widehat{P}}$ is the operator mask.

In plain words, $W_s$ contains all the attack words *w* such that the observation $s'={\widehat{P}}(w)$ can be observed by an operator when there is no attack. The set of exposing words $W_e$ includes all the attack words that are the concatenation of a stealthy word *w* with an event $e \in E_a$ such that *we* is not stealthy. While a stealthy word does not expose the presence of an attacker, an exposing word reveals an attacker, but only at the last step.

The following proposition describes the language and the transition function of an operator observer.

#### Proposition 5

^29^ *Consider a plant G with its observer* $Obs(G) = (B,{E_o},{\delta _{obs}},b_0)$. *Let* $Obs_{opr}(G) = (B_{opr},E_a,\delta _{opr},b_0)$ *be the operator observer constructed using Algorithm 2 in Zhang et al.*^29^. *The following two statements hold*:

- $$L[Obs_{opr}(G)] = W_s \cup W_e$$;
- $\forall w \in L[Obs_{opr}(G)]$: *if* $w \in W_s$, *then* $\delta _{opr}^*(b_0,w)=\delta _{obs}^*[b_0,{\widehat{P}}(w)]$; *if* $w \in W_e$, *then* $\delta _{opr}^*(b_0,w)=b_{\emptyset }$. $\Box$

The formal proof of the above proposition can be found in Zhang et al.^29^ (Proposition 2 therein). The language of an operator observer $L[Obs_{opr}(G)]$ is equal to the union of the set of stealthy words $W_s$ and the set of exposing words $W_e$.

If $w \in W_s$, from the initial state $b_0$, the state reached in $Obs_{opr}(G)$ by executing *w* is equal to the state reached in *Obs*(*G*) by executing ${\widehat{P}}(w)$. It follows from the fact that in $Obs_{opr}(G)$ events in $E_-$ are self-loops, and $Obs_{opr}(G)$ updates its states the same way in case of event $e \in E_{ins}$ and the corresponding event $e_+$. If $w \in W_e$, then state $b_\emptyset$ is reached by executing *w* in $Obs_{opr}(G)$. This means that the attacker is exposed when the attack word $w \in W_e$ is generated.

### Joint estimator

In this subsection we recall the notion of joint estimator and present a characterization of its language and transition function.

#### Definition 6

Given a plant *G* with set of compromised events $E_{com}$. Let $Obs_{att}(G) = (B,E_a,\delta _{att},b_0)$ be the attacker observer, and $Obs_{opr}(G)= (B_{opr},E_a,\delta _{opr},b_0)$ be the operator observer. A joint estimator is a DFA, defined as $A=(R,E_a,\delta _a,r_0)=Obs_{att}(G) \parallel Obs_{opr}(G)$, where

- $R=\{r=(b,{\overline{b}}_a) \ | \ b \in B, {\overline{b}}_a \in B_{opr}\}$ is the set of states,
- $E_a=E_o \cup E_+ \cup E_-$ is the alphabet,
- the transition function $\delta _a[(b,{\overline{b}}_a),e]=[\delta _{att}(b,e),\delta _{opr}({\overline{b}}_a,e)] \text { if } e \in \Gamma _{att}(b) \cap \Gamma _{opr}({\overline{b}}_a)$. Note that, we use $\Gamma _{att}(b)$ (resp., $\Gamma _{opr}({\overline{b}}_a)$) to denote the set of active events at state *b* (resp., ${\overline{b}}_a$) in $Obs_{att}(G)$ (resp., $Obs_{opr}(G)$),
- $r_0=(b_0,b_0)$ is the initial state.  $\square$

We point out that, in the joint estimator *A*, if there exist unreachable states from the initial state $r_0$, then such states should be removed.

For a generic state $r=(b,{\overline{b}}_a)$ of the joint estimator *A*, its first element characterizes the state estimation of the attacker according to the correct observation, and its second element characterizes the state estimation of the operator according to the corrupted observation. Since the attacker observer and the operator observer have the same alphabet $E_a$, according to the definition of concurrent composition, at state $r=(b,{\overline{b}}_a)$, event *e* can occur only if such an event is active at state *b* of $Obs_{att}(G)$, and at state ${\overline{b}}_a$ of $Obs_{opr}(G)$ simultaneously.

The following theorem characterizes the language and the transition function of the joint estimator *A*.

#### Theorem 7

^29^ *Given a plant G with its observer* $Obs(G) = (B,{E_o},{\delta _{obs}},b_0)$, *let* $W_s$ *and* $W_e$ *be the sets of stealthy words and exposing words, and* $A=(R,E_a,\delta _a,r_0)$ *be the joint estimator. The following statements hold*:

- $$L(A) = L({{\mathscr {F}}},G) \cap \left( W_s \cup W_e \right)$$;
- $$\forall s \in P[L(G)]$$, $$\forall f \in {{\mathscr {F}}}$$ *with* $$w=f(s) \in E_a^*$$:
  (i) if $$w \in W_s$$, *then* $$\delta _a^*(r_0,w)=(b_a,{\overline{b}}_a)$$ $$\Longleftrightarrow$$ $$\delta _{obs}^*(b_0,s)=b_a$$, $$\delta _{obs}^*[b_0,{\widehat{P}}(w)]={\overline{b}}_a$$;
  (ii) if $w \in W_e$, *then* $\delta _a^*(r_0,w)=(b_a,b_\emptyset )$ $\Longleftrightarrow$ $\delta _{obs}^*(b_0,s)=b_a$, $\delta _{obs}^*[b_0,{\widehat{P}}(w)]$ *is not defined*. $\Box$

The formal proof of Theorem 7 can be found in Zhang et al.^29^ (Theorem 1 therein). Since the joint estimator *A* is obtained as the concurrent composition of the attacker observer $Obs_{att}(G)$ and the operator observer $Obs_{opr}(G)$ that have the same alphabet $E_a$, then the language of the joint estimator *L*(*A*) is equal to the intersection of their languages. The proof of item (b) follows from Proposition 3, Proposition 5, and the definition of concurrent composition.

## Problem statement

In this paper we want to provide a tool to establish if an attack function exists, which satisfies two main properties, namely stealthiness and harmfulness with respect to a given misleading relation. Such properties can be formalized as follows.

### Definition 8

Consider a plant $G=(X,E,\delta ,x_0)$ with its observer $Obs(G)=(B,E_o,\delta _{obs},b_0)$. An attack function *f* is said to be harmful w.r.t. a misleading relation ${\mathscr {R}} \subseteq 2^X \times 2^X$ if $\exists s \in P(L(G))$ with $s'={\widehat{P}}(f(s))$ such that $({\mathscr {C}}(s),{\mathscr {C}}(s')) \in {\mathscr {R}}$, where ${\mathscr {C}}(s)= \delta _{obs}^*(b_0,s)$ (resp., ${\mathscr {C}}(s')= \delta _{obs}^*(b_0,s')$) is the set of states consistent with observation *s* (resp., $s'$). $\square$

In words, an attack function is harmful if there exists an observation *s* that can be altered into a corrupted observation $s'$ such that the pair of the sets of consistent states belongs to the misleading relation ${\mathscr {R}}$.

### Definition 9

Consider a plant *G* with attack language *L*(*f*, *G*), let *P* be the natural projection, and ${\widehat{P}}$ be the operator mask. An attack function *f* is said to be *stealthy* if ${\widehat{P}}(L(f,G)) \subseteq P(L(G))$. $\square$

In simple words, an attack function is stealthy if the set of words that an operator may observe when the plant is under attack is included in the set of words that the operator may observe when no attack happens. This guarantees that the operator does not realize that the plant is under attack.

To clarify the motivation of introducing the misleading relation ${\mathscr {R}}$, we present the following example.

### Example 10

An operator monitors the plant $G=(X,E,\delta ,x_0)$ to determine if a state in the set of critical states $X_{cr}$ is reached in order to activate protective actions for the plant. An attacker corrupts the operator’s observation preventing it from realizing when a critical state is reached.

The above problem can be defined by a misleading relation ${\mathscr {R}} = \{ (X_1,X_2) \ | \ X_1 \cap X_{cr} \ne \emptyset \text{ and } X_2 \cap X_{cr} = \emptyset \}$, i.e., there exists at least one word $s \in P(L(G))$ such that ${\mathscr {C}}(s) \cap X_{cr} \ne \emptyset$ (indicating that a critical state may have been reached), which can be corrupted into an observation $s' \in E_o^*$ such that ${\mathscr {C}}(s') \cap X_{cr} = \emptyset$ (implying that the operator evaluates that the plant is not in a critical state).

If a critical state has been reached, but the operator does not realize it, then the operator activates no protective actions, and the system will be seriously damaged. $\square$

Consider a plant *G* with set of compromised events $E_{com}$, and a misleading relation ${\mathscr {R}} \subseteq 2^X \times 2^X$. The main contribution of this work is that of constructing an automaton, called supremal stealthy joint subestimator, which contains all the possible attacks that an attacker can carry out during the evolution of the system, while guaranteeing stealthiness. A procedure to compute a stealthy attack function based on the supremal stealthy joint subestimator is proposed. Then it is shown how such a structure allows one to determine if a harmful and stealthy attack function exists. This is not only useful to the attacker, but also to the operator. Indeed, it can be used to evaluate if the system is robust to attacks in the considered setting.

## Supremal stealthy joint subestimator

In this section we first construct the attacker observer $Obs_{att}(G)$, operator observer $Obs_{opr}(G)$, and joint estimator *A*. Then, we show how a joint estimator *A* should be appropriately trimmed to ensure that all the actions that an attacker may implement (erase or insert events) based on it, guarantee stealthiness. The DFA resulting from the trimming operation is called *supremal stealthy joint subestimator*.

### Example 11

**Figure 2:**
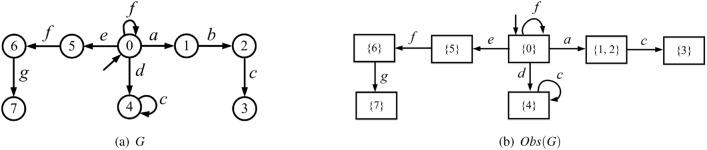
(**a**) A plant *G*; (**b**) its observer *Obs*(*G*).

**Figure 3:**
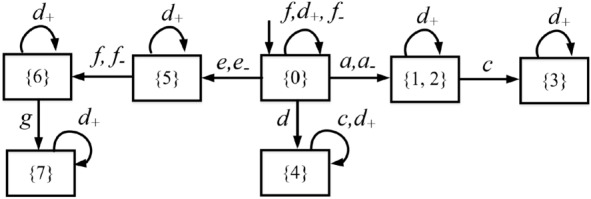
Attacker observer in Example 11 for the plant in Fig. 2.

Consider a plant modeled by a partially-observed DFA $G =({X,E,\delta ,{x_0}})$ in Fig. 2(a). Let $E_o=\{a,c,d,e,f,g\}$ and $E_{uo} = \{b\}$. The observer of the plant is depicted in Fig. 2(b). We assume that $E_{ins}=\{d\}$ and $E_{era}=\{a,e,f\}$. The attacker observer $Obs_{att}(G)$ is sketched in Fig. 3.

Since $a \in E_{era}$, and there exists a transition labeled *a* from state $\{0\}$ to state $\{1,2\}$ in the observer *Obs*(*G*), we add transitions labeled *a* and $a_-$ from state $\{0\}$ to state $\{1,2\}$ in the attacker observer $Obs_{att}(G)$. Similar discussions can be used to clarify the other transitions labeled $e_-$ and $f_-$. Since $d \in E_{ins}$, we add self-loops labeled $d_+$ at all the states of $Obs_{att}(G)$. $\square$

### Example 12

**Figure 4:**
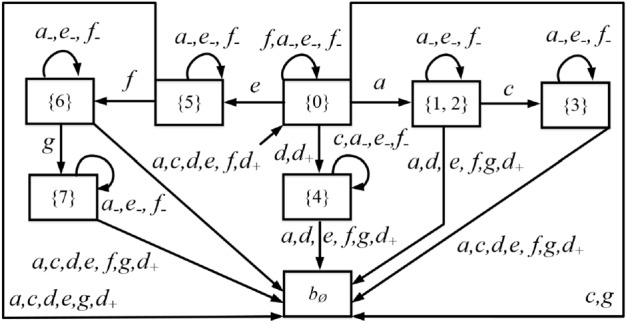
Operator observer in Example 12 for the plant in Fig. 2.

Consider again the plant in Fig. 2. Assume that $E_{ins}=\{d\}$ and $E_{era}=\{a,e,f\}$. The operator observer $Obs_{opr}(G)$ is shown in Fig. 4.

First, since $d \in E_{ins}$, and there exists a transition labeled *d* from state $\{0\}$ to state $\{4\}$ in the observer *Obs*(*G*), we add transitions labeled *d* and $d_+$ from state $\{0\}$ to state $\{4\}$ in $Obs_{opr}(G)$.

Then, since $a,e,f \in E_{era}$, we add self-loops labeled $a_-$, $e_-$, and $f_-$ at all the states of $Obs_{opr}(G)$. Finally, all the missing transitions are added to state $b_\emptyset$ that has no output arc.  $\square$

### Example 13

**Figure 5:**
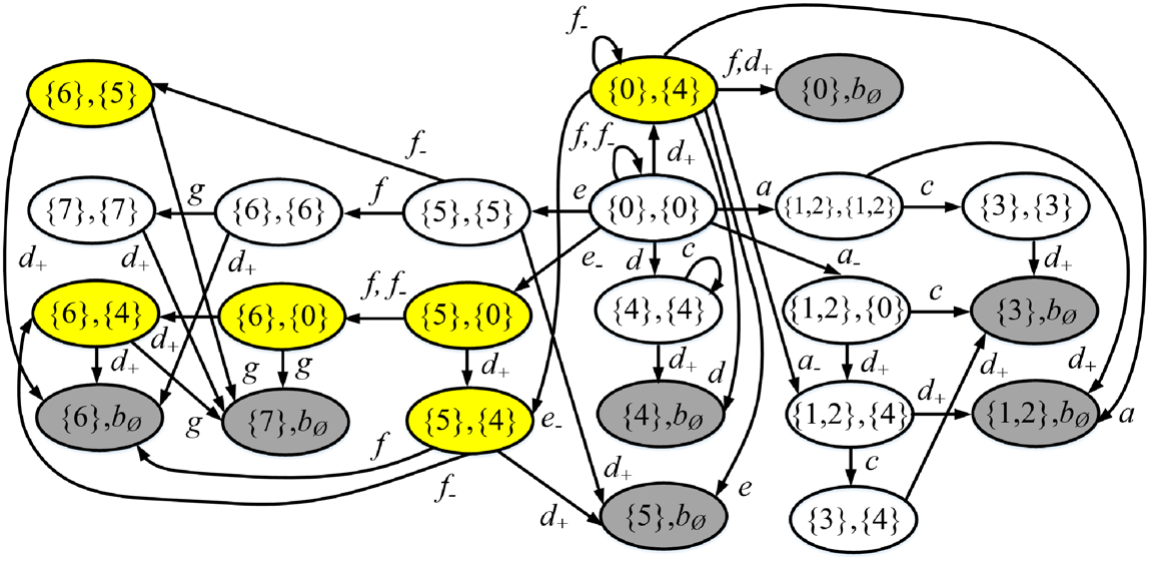
Joint estimator *A* in Example 13.

Review the plant *G* in Example 11, its attacker observer $Obs_{att}(G)$ and operator observer $Obs_{opr}(G)$ are depicted in Figs. 3 and 4, respectively. The joint estimator $A= Obs_{att}(G) \parallel Obs_{opr}(G)$ is sketched in Fig. 5 (the reasons for highlighting the states in different colours will be discussed later).

At the initial state $(\{0\},\{0\})$, event $a \in E_{era}$ may occur in the plant *G*, corresponding to transition $\delta _a[(\{0\},\{0\}),a]=(\{1,2\},\{1,2\})$. In such a case, both the first and the second element of the state are updated since both the attacker and the operator realize the occurrence of such an observable event of *G*. On the other hand, if the attacker erases *a*, this corresponds to transition $\delta _a[(\{0\},\{0\}),a_-]=(\{1,2\},\{0\})$. In this way, only the first element is updated because the operator cannot observe events in $E_-$. In addition, the attacker may also insert $d_+$ before the occurrence of a real event of the plant, corresponding to transition $\delta _a[(\{0\},\{0\}),d_+]=(\{0\},\{4\})$. In such a case, only the second element is updated since the attacker realizes that $d_+$ is a fake event. Similar discussions can be used to clarify the other states and transitions. $\square$

### Definition 14

Given a joint estimator $A=(R,E_a,$ $\delta _a,r_0)$ we define the set of exposing states as $R_e:=\{r=(b_a , {\overline{b}}_a)\in R \ | \ {\overline{b}}_a = b_\emptyset \}$ and the set of stealthy states as $R_s= R \setminus R_e$. $\square$

An attack word leading to an exposing state reveals the presence of an attacker to an operator observing the system’s evolution. Note, however, that there may exist stealthy states from which an exposing state is necessarily reached following a particular evolution of the plant.

### Example 15

Consider the joint estimator *A* in Fig. 5 already discussed in Example 13. When the stealthy state $(\{6\},\{5\})$ is reached, the plant is in state $\{6\}$. At this point, event $g \in E_o \setminus E_{era}$ may occur in the plant. Since the attacker cannot erase event *g*, then the exposing state $(\{7\},b_\emptyset )$ is reached. The attacker may try to preempt the occurrence of event *g* inserting an event in $E_+=\{d_+\}$. However, from $(\{6\}, \{5\})$ inserting such an event also yields exposing state $(\{6\},b_\emptyset )$. $\square$

Consider function $g: 2^{R_s} \rightarrow 2^{R_s}$ defined for all $R' \subseteq R_s$ as follows:

10 $$\begin{aligned} g(R') =g_1(R') \cup g_2(R') \end{aligned}$$

where

11 $$\begin{aligned} \begin{array}{ll} g_1(R') = \left\{ r \in R' \mid \right. {{\textbf {if}} } e \in E_o \ {{\textbf {and}}} \ \delta _a(r,e) \in R \setminus R', {{\textbf {then}}} \ \left. e \in E_{era} \ {{\textbf {and}}} \ \delta _a(r,e_-) \in R' \right\} . \end{array} \end{aligned}$$

and

12 $$\begin{aligned} \begin{array}{ll} g_2(R') = \left\{ r \in R' \setminus g_1(R') \mid \right. (\exists w_+ \in E_{+}^*) \ [\delta _a(r,w_+) \in g_1(R') \ {{\textbf {and}}} \left. (\forall w \prec w_+) \ \delta _a(r,w) \in R'] \right\} . \end{array} \end{aligned}$$

In words, the set $g(R') \subseteq R'$ is the set of states from which a suitable attacker decision can prevent leaving $R'$ and includes states belonging to two different sets: $g_1(R')$ and $g_2(R')$. Set $g_1(R')$ includes the states of $R'$ such that, if there exists an observable event *e* whose occurrence leads outside $R'$, then the attacker may cancel it to remain in $R'$. Set $g_2(R')$ includes the states of $R'$ that do not belong to $g_1(R')$, from which it is possible to reach a state in $g_1(R')$ inserting a word $w_+$, and all the states visited generating it belong to $R'$.

A fixed-point of *g* is a set $R_{fix} \subseteq R_s$ such that $g(R_{fix}) = R_{fix}$.

The following theorem shows that function *g* in Eq. (10) has a *supremal* (i.e., unique maximal) non-empty fixed point.

### Theorem 16

Consider a joint estimator $A=(R,E_a,$ $\delta _a,r_0)$ with set of stealthy states $R_s$. Let $g:2^{R_s}\rightarrow 2^{R_s}$ be the function defined for all $R'\subseteq R_s$ as in eq. (10). Function *g* has a non-empty supremal fixed point, denoted in the following as $R_{sf}$.

### Proof

We preliminarily observe that function *g* is monotone, i.e., by definition for all $R' \subseteq R''$ it holds that $g(R') \subseteq g(R'')$. Thus according to Tarski’s fixed-point theorem^32^ function *g* has a *unique maximal fixed-point*, i.e., a *supremal fixed-point*, that we denote as $R_{sf}$ and that can be computed as

13 $$\begin{aligned} R_{sf} = \bigcap _{k \ge 0} g^k(R_s) \end{aligned}$$

in at most $|R_s|$ iterations^33^.

In addition, one can easily verify that, for all joint estimators *A*, the set of states reachable without any attack

14 $$\begin{aligned} R_0 = \{(b_a , {\overline{b}}_a)\in R \ | \ b_a = {\overline{b}}_a \} \subseteq R_s \end{aligned}$$

is a fixed-point of *g* since the occurrence of an event $e \in E_o$ does not lead out of this set: this ensures that $R_{sf} \supseteq R_0$ is not empty. $\square$

In the following, the supremal fixed-point $R_{sf}$ of function *g* is called *strongly stealthy region*, and it can be computed using Eq. (13). Set $R_w= R \setminus R_{sf}$ is called *weakly exposing region*.

### Example 17

Consider again the partially-observed plant $G = \left( {X,E,\delta ,{x_0}} \right)$ in Fig. 2, where $E_o=\{a,c,d,e,f,g\}$, $E_{uo} = \left\{ b \right\}$, $E_{ins}=\{d\}$, and $E_{era}=\{a,e,f\}$. The joint estimator *A* is shown in Fig. 5.

Here, exposing states are highlighted in gray, while states in $R_w$ that are not exposing are highlighted in yellow.

To clarify how non-exposing states are added to $R_w$, let us consider state $(\{6\},\{5\})$. There exists a transition labeled $g \in E_o \setminus E_{era}$ that leads from $(\{6\},\{5\})$ to a state in $R_w$ (in such a case the exposing state is $(\{7\}, b_\emptyset )$). This is equivalent to say that $(\{6\},\{5\})$ does not belong to $g_1(R \setminus R_w)$ [Eq. (11)]. In addition, $(\{6\},\{5\})$ also does not belong to $g_2(R\setminus R_w)$ [Eq. (12)] because from such a state it is not possible to reach a state in $g_1(R \setminus R_w)$ adding a sequence of events in $E_+$. Hence $(\{6\},\{5\}) \notin g(R \setminus R_w)$. Once state $(\{6\},\{5\})$ is reached, following the evolution of the plant, an exposing state ($(\{6\},b_\emptyset )$ or $(\{7\},b_\emptyset )$) will be necessarily reached.

We notice that state $(\{0\},\{4\})$ should also be added to $R_w$ because there exists a transition labeled $d \notin E_{era}$ that leads to state $(\{4\}, b_\emptyset )$, and there does not exist a transition labeled $d_+$ that leads it to a state not in $R_w$. However, at state $(\{0\},\{4\})$, if event *a* occurs, the attacker can erase it leading to state $(\{1,2\},\{4\})$ that does not belong to $R_w$. This means that, at state $(\{0\},\{4\})$, if event *a* occurs, then the attacker can erase it to remain stealthy; if event *d* occurs, then the attacker is discovered, i.e., the stealthiness of the attacker depends on the future evolution of the plant.

At the first iteration of Eq. (13), states $(\{0\},\{4\})$, $(\{6\},\{4\})$, and $(\{6\},\{5\})$ are added to $R_w$; at the second iteration, states $(\{5\},\{4\})$ and $(\{6\},\{0\})$ are added; finally at the third iteration, state $(\{5\},\{0\})$ is added. $\square$

### Definition 18

Consider a joint estimator $A=(R,E_a,\delta _a,r_0)$ with set of stealthy states $R_s$. Let $g:2^{R_s}\rightarrow 2^{R_s}$ be the function defined in Eq. (10) and $R_{sf}$ be its supremal fixed point. The DFA ${\widehat{A}}=({\widehat{R}},E_a,{\widehat{\delta }}_a,r_0)$ called supremal stealthy joint subestimator of *A*, is obtained from *A* in two steps:

- Let $A'$ be the automaton obtained removing from *A* all states in $R \setminus R_{sf}$ and their input and output arcs.
- Let ${\widehat{R}}$ be the set of reachable states in $A'$ and let ${\widehat{A}}$ be the automaton obtained from $A'$ removing all states that are not in ${\widehat{R}}$ and their input and output arcs. 

$$\square$$

In simple words ${\widehat{A}}=({\widehat{R}},E_a,{\widehat{\delta }}_a,r_0)$ is obtained trimming *A* first removing all states that are not in $R_{sf}$ and then removing all states that are unreachable from the initial state.

### Example 19

**Figure 6:**
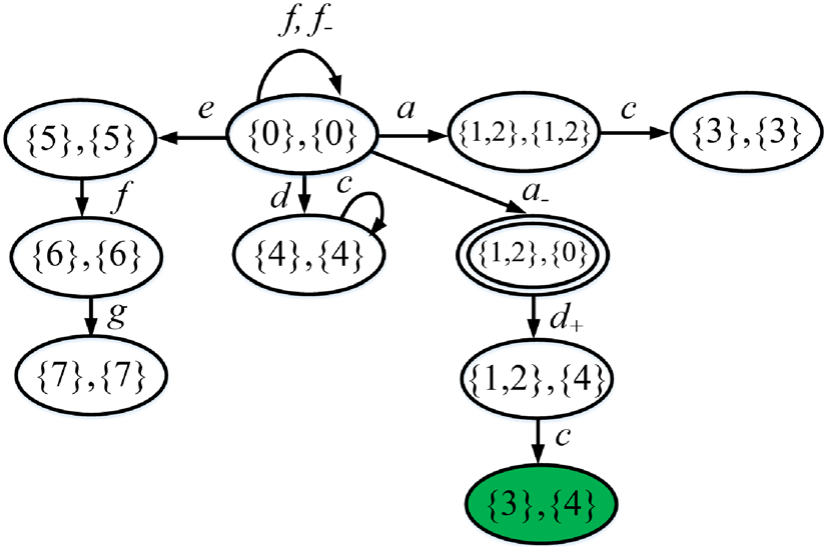
Supremal stealthy joint subestimator ${\widehat{A}}$ in Example 19.

Consider again the plant $G = \left( {X,E,\delta ,{x_0}} \right)$ in Fig. 2 and its joint estimator *A* in Fig. 5, as discussed in Example 17. The corresponding supremal stealthy joint subestimator is shown in Fig. 6. The reason for colouring in green state $(\{3\},\{4\})$ and marking state $(\{1,2\},\{0\})$ with a double circle will be discussed in the following section. $\square$

### Proposition 20

*The language* $L({\widehat{A}})$ *of the supremal stealthy joint subestimator is the set of all attack words that are stealthy and can be kept stealthy by a proper action of the attacker, regardless of the future evolution of the plant.*

### Proof

By definition, a fixed-point of function *g* is a set of stealthy states of the joint estimator that the attacker can make invariant by choosing a suitable action. Correspondingly, the words generated by evolutions that remain within such a set can be kept stealthy by the attacker. The fact that set $L({\widehat{A}})$ contains all such words follows from the fact that ${\widehat{R}}$ is the set of reachable states that belong to the supremal fixed-point of *g*. $\square$

Now we discuss the complexity of constructing the supremal stealthy joint subestimator ${\widehat{A}}$.

Let *G* be a plant with set of states *X*, the observer of the plant *Obs*(*G*) has at most $2^{|X|}$ states, the attacker observer $Obs_{att}(G)$ has at most $2^{|X|}$ states, and the operator observer $Obs_{opr}(G)$ has at most $2^{|X|}+1$ states. As a result, the joint estimator $A=Obs_{att}(G) \parallel Obs_{opr}(G)$ has at most $2^{|X|} \cdot (2^{|X|}+1)$ states.

In addition, testing if a state of a joint estimator $r \in g(R \setminus R_w)$ has linear complexity in the size of *A*. Thus, the complexity of constructing ${\widehat{A}}$ is $O(2^{4|X|})$.

## Computing stealthy and harmful attack function

This section consists of three subsections. In “Preempting states” subsection we show how to identify a subset of states of the supremal stealthy joint subestimator, called *preempting states*. In “Selection of a stealthy attack function” subsection we show how to select a stealthy attack function from the supremal stealthy joint subestimator. Finally, in “Existence of a stealthy and harmful attack function w.r.t. a relation ${\mathscr {R}}$” subsection we discuss how the existence of a stealthy and harmful attack function can be verified by means of the previous subestimator.

### Preempting states

In this subsection we define a subset of the states of the supremal stealthy joint subestimator ${\widehat{A}}$, called *preempting states*, which are needed to define a procedure to select a stealthy attack function from ${\widehat{A}}$.

#### Definition 21

Consider a joint estimator $A=(R,E_a,\delta _a,r_0)$ with supremal stealthy joint subestimator ${\widehat{A}}=({\widehat{R}},E_a,{\widehat{\delta }}_a,r_0)$. The set of preempting states of ${\widehat{A}}$ is

15 $$\begin{aligned} \begin{array}{ccl} {\widehat{R}}_p = \{ r \in {\widehat{R}} \ \mid (\exists e \in E_o) \ \delta _a(r, e) \in R \setminus {\widehat{R}} \ \wedge \ (e \not \in E_{era} \ \vee \ \delta _a(r, e_-) \in R \setminus {\widehat{R}} ) \ \}. \\ \end{array} \end{aligned}$$

Define the set of non-preempting states of ${\widehat{A}}$ as

16 $$\begin{aligned} {\widehat{R}}_{np}={\widehat{R}} \setminus {\widehat{R}}_p. \end{aligned}$$

$$\square$$

Recalling the definition of function $g_2$ given in eq. (12), it is straightforward to observe that ${\widehat{R}}_p = g_2({\widehat{R}})$.

Note that a state $r \in {\widehat{R}}_p$ (namely a state of the supremal stealthy joint subestimator ${\widehat{A}}$) is preempting if there exists an observable event *e* in the original joint estimator whose occurrence (even if erased) leads out of ${\widehat{R}}$ and this may eventually lead to expose the attacker. However, the occurrence of such observable event *e* can be preempted inserting a suitable sequence of events in $E_+$ so as to reach a non-preempting state.

#### Example 22

Recall the partially-observed plant $G = \left( {X,E,\delta ,{x_0}} \right)$ in Fig. 2(a) with joint estimator *A* in Fig. 5 and supremal stealthy joint subestimator in Fig. 6. Looking at Fig. 5 we realize that $(\{1,2\},\{0\})$ is a preempting state because the occurrence of event *c*, which cannot be erased, yields exposing state $(\{3\},b_\emptyset )$. The preempting state is marked with a double circle in Fig. 6. Once state $(\{1,2\},\{0\})$ is reached, event $d_+$ should be inserted to reach a state that is not preempting.  $\square$

### Selection of a stealthy attack function

In this subsection we show how an attacker may determine a stealthy attack function $f^s$ given a supremal stealthy joint subestimator. This can be done associating to each possible observation produced by the plant a suitable attack word.

The proposed approach is summarized in the following steps. Note that here, given a state $r\in {\widehat{R}}$, we denote as $\Gamma _{A}(r) \subseteq E_a$ the set of events enabled at *r* in ${\widehat{A}}$. Furthermore, we denote as ${{\mathscr {W}}}_+(r) \subseteq E_+^*$ the set of words that can be generated in ${\widehat{A}}$ starting from *r* and executing a sequence of events $w_+\in E_+^*$ that lead to a non-preempting state, namely,

17 $$\begin{aligned} {{\mathscr {W}}}_+(r)=\{w_+ \in E_+^{*} \ \mid \ {\widehat{\delta }}_a^*(r,w_+)=r', \ r' \in {\widehat{R}}_{np} \}. \end{aligned}$$

#### Procedure 1

(Compute a stealthy attack function $f^s$ from a supremal stealthy joint subestimator ${\widehat{A}}=({\widehat{R}},E_a,{\widehat{\delta }}_a,r_0)$).

1. Let $$s=\varepsilon$$.
2. Select a sequence $$w_+\in {{\mathscr {W}}}_+(r_0)$$.
3. Let $$f^s(s)=w_+$$.
4. Let $$r={\widehat{\delta }}_a^*(r_0,w_+)$$.
5. Wait for the system to generate a new event $e\in E_o$.
6. $${{\mathscr {E}}} = \emptyset$$.
7. If $$e \in \Gamma _{\widehat{A}}(r)$$ then $${{\mathscr {E}}} = {{\mathscr {E}}} \cup \{ e \}$$.
8. If $$e_- \in \Gamma _{\widehat{A}}(r)$$ then $${{\mathscr {E}}} = {{\mathscr {E}}} \cup \{ e_- \}$$.
9. Select an event $e' \in {{\mathscr {E}}}$ and a sequence $w_+\in {{\mathscr {W}}}_+({\widehat{\delta }}_a(r,e'))$ and let $w=e' w_+$.
10. Let $$f^s(se)=f^s(s)w$$.
11. Let $$s=se$$.
12. Let $$r={\widehat{\delta }}_a^*(r,w)$$.
13. Goto Step 5.

The above procedure can be explained as follows. If no event occurs in the plant, the attacker can insert a word $w_+ \in E_+^{*}$, provided that the state reached executing *w* in ${\widehat{A}}$ is in ${\widehat{R}}_{np}$, namely it is not a preempting state. Note that in general the choice of $w_+$ is not unique. Indeed, in Step 2 we select one $w_+$ in the set ${{\mathscr {W}}}_+(r_0)$, which in general is not a singleton. In Step 3 we update accordingly function $f^s$, and in Step 4 we compute the new current state of ${\widehat{A}}$, denoted as *r*.

We then wait for the system to generate a new observable event *e* (Step 5). In this case a new set ${{\mathscr {E}}}$ is defined and it is initialized at the empty set. As specified in Steps 7 and 8, such a set may contain the event *e*, if *e* is enabled at *r*. In addition, it may contain the event $e_-$, if $e_-$ is enabled at *r*. At Step 9 one event $e^{\prime }\in {{\mathscr {E}}}$ is selected, as well as one word $w_+\in {{\mathscr {W}}}_+({\widehat{\delta }}_a(r,e^{\prime }))$. Finally, the corrupted word *w* is defined as the concatenation of $e^\prime$ and $w_+$.

Then, function $f^s$ is updated accordingly (Step 10), as well as the observation *s* (Step 11) and the current state *r* of ${\widehat{A}}$ (Steps 12). The procedure goes ahead (Step 13) when a new observable event is generated, starting again from Step 5.

As discussed in the above procedure, the key feature in selecting a stealthy attack function is that of choosing, from the supremal stealthy joint subestimator, attack words that do not end in a preempting state.

#### Example 23

Review the plant $G = \left( {X,E,\delta ,{x_0}} \right)$ in Fig. 2(a). We show how to compute a stealthy attack function on the basis of the supremal stealthy joint subestimator ${\widehat{A}}$ which is sketched in Fig. 6. At the initial state $(\{0\},\{0\})$, since $a_- \in \Gamma _{A}((\{0\},\{0\}))$, the attacker chooses such an action, corresponding to the transition $\delta _a[(\{0\},\{0\}),a_-]=(\{1,2\},\{0\})$. Since $(\{1,2\},\{0\})$ is a preempting state, the attacker does not stay there. It chooses to insert $d_+$ leading to state $(\{1,2\},\{4\})$. In this way, the correct observation *a* is altered into the corrupted observation *d*. $\square$

#### Proposition 24

*Consider a plant G under attack and let* $f:P(L(G))\rightarrow E_a^*$ *be an attack function.*

Function *f* is stealthy if and only if for all $s \in P(L(G))$ the attack word *f*(*s*) can be computed by Procedure 1.

#### Proof

We denote by $A=(R,E_a,\delta _a,r_0)$ the joint estimator of *G* with set of stealthy states $R_s$. We also denote by ${\widehat{A}}=({\widehat{R}},E_a,{\widehat{\delta }}_a,r_0)$ the supremal stealthy joint subestimator computed by *A* using Definition 18.

(If) By construction ${\widehat{R}} \subseteq R_s \subseteq R$, i.e., language $L({\widehat{A}})$ only contains stealthy words and thus any word computed by Procedure 1 is stealthy. In addition, a word computed by the procedure yields a non-preempting state $r \in {\widehat{R}}$, i.e., $r \in g_1({\widehat{R}})$ using the notation of Eq. (11). This means that for any observable event $e \in E_o$ that the plant can generate after *s*, the procedure will compute a new word *f*(*se*) still in $L({\widehat{A}})$ and thus function *f* is stealthy.

(Only if) Assume that for a given attack function *f* there exists an observation $s \in P(L(G))$ such that *f*(*s*) cannot be computed by Procedure 1. Two cases are possible.

- If $f(s) \in L({\widehat{A}})$, then *f*(*s*) yields a preempting state and there exists some observable event $e \in E_o$ such that $f(se) \in L(A) \setminus L({\widehat{A}})$, i.e., *f*(*se*) yields in *A* a state not in ${\widehat{R}}$.
- If $f(s) \in L(A) \setminus L({\widehat{A}})$, then *f*(*s*) yields in *A* a state not in ${\widehat{R}}$.

This means that attack function *f* produces an attack word not in $L({\widehat{A}})$ and hence, by Proposition 20, it cannot be stealthy.  $\square$

### Existence of a stealthy and harmful attack function w.r.t. a relation ${\mathscr {R}}$

In this subsection we characterize those cases in which a stealthy attack function that is harmful w.r.t. a certain relation ${\mathscr {R}}$ exists.

#### Proposition 25

*Consider a plant* $G=(X,E,\delta ,x_0)$ *under attack and let* ${\widehat{A}}=({\widehat{R}},E_a,{\widehat{\delta }}_a,r_0)$ *be its supremal stealthy joint subestimator. Given a misleading relation* ${\mathscr {R}} \subseteq 2^X \times 2^X$, *a stealthy and harmful attack function f can be selected iff* ${{\widehat{R}}}_{np} \cap {\mathscr {R}} \ne \emptyset$, *where* ${{\widehat{R}}}_{np}$ *is the subset of non-preempting states in* ${{\widehat{R}}}$.

#### Proof

(If) Assume that, in ${\widehat{A}}$, there exists a state $r \in {{\widehat{R}}}_{np} \cap {\mathscr {R}}$ such that $r= {\widehat{\delta }}_a^*(r_0,w)$ and $w=f(s)$, where $s \in P(L(G))$, and *P* is the natural projection. Since $r \in {{\widehat{R}}}_{np}$, it means that the attack word *w* does not end in a preempting state. Then according to Procedure 1 and Proposition 24, we can conclude that *f* is stealthy.

Since $r=(b_a,{\overline{b}}_a) \in {\mathscr {R}}$, on the basis of Theorem 7, if $w \in W_s$, then $b_a=\delta _{obs}^*(b_0,s)$, and ${\overline{b}}_a=\delta _{obs}^*(b_0,{\widehat{P}}(w))$. This indicates that there exists an observation *s* that can be corrupted into a word $s'={\widehat{P}}(w)$ such that $({\mathscr {C}}(s),{\mathscr {C}}(s') \in {\mathscr {R}}$, where ${\mathscr {C}}(s)=b_a$, ${\mathscr {C}}(s')={\overline{b}}_a$, and ${\widehat{P}}$ is the operator mask. According to Definition 8, it can be concluded that *f* is also harmful.

(Only if) Assume that there exists a stealthy and harmful attack function *f*. Since *f* is stealthy, according to Procedure 1 and Proposition 24, the attack word $w=f(s)$ does not end in a preempting state of ${\widehat{A}}$, thus state $r={\widehat{\delta }}_a^*(r_0,w) \in {{\widehat{R}}}_{np}$.

Since *f* is harmful, on the basis of Definition 8, there exists an observation *s* that can be changed into a corrupted observation $s'$ such that $({\mathscr {C}}(s),{\mathscr {C}}(s') \in {\mathscr {R}}$, where ${\mathscr {C}}(s)= \delta _{obs}^*(b_0,s)$, and ${\mathscr {C}}(s')= \delta _{obs}^*(b_0,s')$. According to Theorem 7, if $w \in W_s$, then state $r={\widehat{\delta }}_a^*(r_0,w)=(b_a,{\overline{b}}_a)$, where $b_a=\delta _{obs}^*(b_0,s)$, ${\overline{b}}_a=\delta _{obs}^*(b_0,s')$, and $s'={\widehat{P}}(w)$. Therefore, state $r \in {\mathscr {R}}$. According to the above discussions, state $r \in {\widehat{R}}_{np} \cap {\mathscr {R}}$, i.e., ${\widehat{R}}_{np} \cap {\mathscr {R}} \ne \emptyset$.

Note that, in the above proofs, we exclude the case that $w \in W_e$ because all the exposing states have been removed from ${\widehat{A}}$. $\square$

#### Example 26

Consider again the partially-observed plant $G = \left( {X,E,\delta ,{x_0}} \right)$ in Fig. 2(a), where $E_o=\{a,c,d,e,f,g\}$ and $E_{uo} = \left\{ b \right\}$. Let the misleading relation ${\mathscr {R}} = \{ (\{3\}, X ) \ | \ X \subseteq \{0,4\} \}$. The supremal stealthy joint subestimator ${\widehat{A}}$ is shown in Fig. 6.

State $(\{3\},\{4\})$, highlighted in green, is a harmful state. When such a state is reached following the attacked observation, the plant is in state $\{3\}$, while the operator thinks it is in state $\{4\}$. In such a case, the attack is harmful. In particular, the harmful attack can be realized by first erasing the occurrence of event *a*, then inserting $d_+$, and finally waiting for the plant to generate event *c*, so that the correct observation $s= ac$ is corrupted to $s'=dc$. $\square$

### Conclusions and future work

The problem of state estimation of discrete event systems under attack has been investigated. The joint estimator, which takes into account the state estimation of the attacker in accordance with the real observation and the state estimation of the operator in accordance with the corrupted observation, is computed as the concurrent composition of two particular structures called the attacker observer and operator observer. By appropriately trimming the joint estimator we obtain the supremal stealthy joint subestimator, which contains all the attacks that keep the attacker stealthy. According to the definition of preempting state, a formal procedure to select a stealthy attack function from such a subestimator is provided. Finally, it is shown how to synthesize a stealthy and harmful attack function w.r.t. the misleading relation ${\mathscr {R}}$.

In the future, on the one hand, we plan to discuss how the proposed procedure may also be useful to an operator, in the case that the system is not robust to attack, to prevent the occurrence of certain events, via a supervisory control law, in order to enforce robustness w.r.t. attack. On the other hand, we intend to consider the case that the attacker only has a partial knowledge of the plant model. It is also interesting to solve the problem considered in this paper using Petri nets, which may provide a more efficient solution.
